# Breaking the barriers: overcoming dementia-related stigma in minority communities

**DOI:** 10.3389/fpsyt.2023.1278944

**Published:** 2023-12-20

**Authors:** Joyce Siette, Anjani Meka, Josefine Antoniades

**Affiliations:** ^1^The MARCS Institute for Brain, Behaviour and Development, Western Sydney University, Westmead, NSW, Australia; ^2^Australian Institute for Health Innovation, Macquarie University, Macquarie Park, NSW, Australia; ^3^National Ageing Research Institute, Affiliate Global and Women’s Health, School of Public Health and Preventive Medicine, Monash University, Royal Melbourne Hospital, Parkville, VIC, Australia

**Keywords:** dementia, stigma, CALD communities, cultural beliefs, language barriers, migration, dementia care, lack of awareness

## Abstract

Dementia is a global health concern that affects individuals irrespective of their cultural or linguistic backgrounds. However, research has long recognized the pronounced stigma associated with dementia, particularly within Culturally and Linguistically Diverse (CALD) communities. This article seeks to summarize the underlying factors contributing to the heightened levels of dementia stigma within CALD communities, through a review of the literature. Our examination shows that cultural beliefs, language barriers, limited awareness, and the impact of migration on perceptions of aging and cognitive decline are contributing factors. Consequently, our analysis highlights the need for tailored, culturally appropriate interventions aimed at mitigating stigma and enhancing dementia care within CALD populations. Our proposed solutions, built on a social-ecological approach, highlights the critical role of collaborative efforts involving policymakers, healthcare providers, community organizations, and CALD community members in fostering a more dementia-inclusive society. This perspective piece aims to shed light on the distinct challenges faced by CALD communities, while advocating for a holistic approach to redefine perceptions and care strategies tailored to these populations.

## Introduction

Stigma surrounding dementia is a pervasive challenge in culturally and linguistically diverse (CALD) communities (1–3). This stigma is complex, exhibits variations among different CALD communities, reflecting the interplay of sociocultural factors (such as beliefs about the etiology of dementia), negative stereotypes associated with individuals living with dementia, and the negative connotations of the terminologies employed (3).

The consequences of dementia-related stigma extend beyond individual experiences and encompass the broader familial and societal landscape. Stigma significantly influences help-seeking behaviors and exerts profound ramifications on the diagnosis and treatment of dementia (3). To address this multifaceted issue within CALD communities, it is imperative to adopt tailored approaches that respect and integrate the specific cultural values and beliefs of these communities, while also confronting and dismantling the societal factors that perpetuate stigma. On the basis of the broader literature on inclusive dementia research both in Australia and internationally (4–6), and the collective experience working with CALD communities in Australia (4, 5, 7), we propose a set of five recommendations aimed at fostering culturally adapted awareness and understanding of dementia, delivering healthcare services that acknowledge cultural nuances, and combating the negative attitudes and stereotypes associated with the condition. These recommendations offer a systematic approach to mitigate dementia-related stigma, ultimately enhancing the wellbeing of CALD individuals and their families.

## Dementia

Dementia has emerged as a significant global health concern, affecting more than 55 million individuals worldwide, with an even greater impact on low- and middle-income countries where over half of diagnosed individuals reside (8). The World Health Organization acknowledges the increasing necessity for worldwide efforts to raise awareness and diminish the stigma surrounding dementia (9). Addressing stigma, especially for health conditions such as dementia, which are sometimes referred to using derogatory terms related to mental instability, presents a significant challenge. However, dementia-related stigma often exacerbates the challenges faced by individuals and their families, especially within CALD communities (10). Understanding the specific factors that underpin stigmatizing attitudes toward dementia across and within specific CALD groups, and working in close collaboration with the target groups is vital to developing strategies.

### Historical origins and stigmatization in public health: a critical examination

The phenomenon of stigmatization in public health has deep historical roots and has been observed across various illnesses, ailments, and personal traits (10–13). In general, stigma is described as a socially humiliating mark of disgrace leading to devaluing of the person in society (1), which includes negative beliefs (stereotypes or prejudice), ignorance, and discriminatory behavior that leads to unequal treatment (9). Stigma can be depicted across three categories: *public* (general public attitudes and beliefs about people with the condition or their family members), *self/personal* (when people internalize these public attitudes and experience negative consequences), and *courtesy* (emotions and behaviors toward family and professionals involved with the person with the condition) (14).

Conditions such as tuberculosis, sexually transmitted diseases, infectious diseases, cancer, and mental illness have carried pervasive stigma throughout history (15–19). The presence of stigma unquestionably serves as a major obstacle in various critical aspects of healthcare, such as receiving timely diagnoses, seeking support following a diagnosis, and disclosing one’s condition to family and friends, especially when dealing with conditions that are stigmatized [e.g., dementia (13)]. These barriers have cascading negative effects on the physical and mental health of individuals, leading to delayed or inadequate access to healthcare, social exclusion, and detrimental psychological consequences (20–23). Further to this, it is not solely the person living with the condition that is impacted by stigma attached to the condition, often there are significant flow-on effects on families and carers (24–27). This can include shame of having a relative with a stigmatizing condition, fear that the illness may run in the families impacting social standing and in some cultures impact marriage prospects for other family members (27).

## Stigma and dementia within CALD communities

While stigma surrounding certain health conditions is prevalent in many societies, dementia carries an exceptional level of societal misconceptions, fear, and discrimination (14, 28–31). Furthermore, CALD communities often face unique cultural and societal factors that contribute to the intensified stigmatization of dementia (31).

Australia has one of the world’s most multicultural populations, with nearly half of the population identifying as culturally and linguistically diverse (CALD), which indicates they were born abroad, have an overseas parent, and/or speak multiple languages (32). In 2021, the top five languages spoken at home other than English were Mandarin, Arabic, Vietnamese, Cantonese, and Punjabi (33). The CALD population often experiences more health concerns (language hurdles, reduced health literacy, and difficulty accessing health care and systems) as well as higher prevalence of chronic diseases such as heart diseases, kidney diseases, lung and mental health conditions and barriers to accessing health services (32). Current data suggests that 28% of persons with dementia in Australia were born in a non-English-speaking country (33), however, this is likely an under-estimation of actual numbers.

The pervasive stigma of dementia in CALD communities has significant implications for dementia care and support (34, 35). Stigma can act as a barrier to accessing timely diagnosis, appropriate treatment, and supportive services (34–36). CALD individuals with dementia may face discrimination, social isolation, and reduced quality of life due to the stigma associated with their condition (1). Furthermore, stigma may lead to a lack of understanding and empathy from healthcare providers and the broader community, resulting in inadequate support and care (37). Understanding the reasons behind the existence of dementia-related stigma in CALD communities is crucial for developing targeted interventions and support systems that effectively address this issue, promote awareness, and foster inclusive and supportive environments for those affected by dementia and their loved ones. Here we present a summary of key issues and how these compound the challenges faced by individuals living with dementia and their families within CALD communities.

### Influence of cultural beliefs and attitudes on dementia perception: role of traditional view of aging

Cultural beliefs and attitudes significantly influence the perception of dementia in CALD communities (38). Traditional views on aging, which vary across cultures, can shape societal attitudes toward dementia (38). In some cultures, aging is revered and respected, while in others, it may be associated with negative connotations or stigma (39). For example, cultures that prioritize intergenerational support may perceive dementia as a personal or familial failure, leading to increased stigma and shame (40, 41). Additionally, collectivist cultures may place a strong emphasis on caregiving within the family, resulting in reluctance to seek external support or professional assistance (42–44).

In general, CALD individuals diagnosed with dementia are likely subjected to stereotypes and negative attitudes regarding dementia status that portray them as a burden on their families or society (45).

In Chinese, Arabic, Vietnamese, and Punjabi communities, dementia is often associated with negative connotations that reflect the cultural understanding of the condition. In Chinese communities, the term “老人痴呆,” which is commonly used and accepted, implies that the condition is senile in nature (i.e., old age retardation) and supports a fatalistic attitude toward dementia, with little focus on prevention or treatment. Whilst there have been changes to adapt this terminology, e.g., “腦退化症” and “認知障礙症” these new phrases recognize that there are barriers that obstruct the brain’s function, however, have yet to be used more widely.

In Arabic-speaking communities, the term dementia does not exist and an alternative phrase “الخرف” is often used and connotates craze and lunacy. For Vietnamese communities, the term “chứng sa sút trí tuệ” focuses on the syndrome of cognitive decline and refers to the condition as a progressive and irreversible decline of cognitive abilities. This can lead to a sense of hopelessness and despair, with little focus on intervention or support.

In Punjabi communities, the term “ਦਿਮਾਗੀ ਕਮਜ਼ੋਰੀ/Dimāgī kamazōrī” reflects the loss of a fundamental aspect of an individual’s identity. This can lead to feelings of shame, embarrassment, or isolation. In general, we find that common terms for dementia focus on the condition as a “normal part of aging,” “insanity,” “crazy,” and “mental illness.” The disease can also label as “evil,” “an act of God,” “a divine test,” “punishment,” “one’s bad karma,” or “the work of spirits and demons” (34, 46–48). Such terminologies are likely to contribute to feelings of humiliation, dread, and disgrace and causes individuals to avoid discussing their symptoms and conceal them (34). In these groups, family stigma also plays a crucial role in supporting misbeliefs and enabling inaction and or concealment.

The limited understanding and awareness around dementia among CALD populations may significantly contribute to the perpetuation of stigma (37). Dementia is often viewed as a natural consequence of aging, rather than a medical condition that requires treatment and care (49). Many CALD individuals and their families have limited understanding of dementia symptoms, causes, and available support systems (1, 50–52). Cultural taboos and misconceptions further contribute to the stigma associated with dementia (1). Fear of discrimination/social isolation and judgment by community also discourage CALD individuals from seeking medical help, leading to a delayed diagnosis, uptake of appropriate treatment and support services (53).

## Language barrier and communication challenges

Language barriers pose significant challenges for CALD individuals with dementia and their families (54). Limited English proficiency has a significant impact on individuals’ ability to access information about dementia, navigate available services, and access potential support networks (55). Miscommunication or misunderstandings during healthcare encounters can contribute to frustration, confusion, and increased stigma (56). In such instances, the involvement of translators or cultural brokers in these situations is significant in promoting successful communication and overcoming language barriers (57). Moreover, these individuals possess a deep understanding of the social norms that must be delicately negotiated within healthcare interactions (58, 59). The inclusion of culturally competent translators within healthcare teams may facilitate the navigation of the healthcare system, enable CALD patients to articulate their issues, and facilitate access to suitable healthcare services (59).

## Current evidence on dementia stigma reduction strategies

In order to address the stigma surrounding dementia in CALD communities, several research bodies have advocated that firstly awareness and understanding of the disease should be improved (36, 37, 60, 61). This can be achieved through tailored education and outreach programs that target CALD communities using culturally sensitive and appropriate messaging, as well as through culturally sensitive healthcare services that consider the unique needs and values of CALD individuals (2, 37, 62). Healthcare providers should be cognizant of the stigma and fear that is attached to diseases such as dementia and be trained to broach these highly sensitive and confronting conversations in ways that aim to ameliorate fear and stigma for those who have been diagnosed with the disease and their carers (63).

In 2008, campaigns to educate the public, reduce discrimination, and promote awareness of dementia were initiated in culturally diverse, developed countries such as United Kingdom, United States and Scotland (14). These campaigns led to initiatives typically targeting three strategies: *education* (dispel misconceptions and update knowledge), *contact* (to encourage people with health issues to open out to one another) and *mixed* (a combination of education and contact). These strategies however are not new, with prior stigma reduction programs toward specific health conditions being generally met with success (64–67). For example, HIV/AIDS stigma reduction programs have been effective in increasing knowledge and understanding of the disease, reducing discriminatory attitudes toward those living with HIV/AIDS, and improving the quality of life for affected individuals (11). Similarly, anti-stigma programs aimed at reducing mental illness stigma have also shown promise, with reported significant reductions in negative attitudes toward mental illness following intervention (68). However, success often depends on the nature of the program, the target population, the cultural context, and investment placed in the campaign.

Recent work on reducing stigma related to dementia has been met with varying degrees of success. These interventions primarily consist of presentations, theater films, curriculum-based education, intergenerational storytelling, visual arts, and performing arts, with the key components of these interventions relating to providing disease facts as well as highlighting the accomplishments of individuals living with dementia (4). However, these programs often lack generalisability, with only dementia care workers, family carers, healthcare college students, and healthcare professionals being targeted (4). Further, there is a lack of rigorous evaluation methods, and these interventions were only from developed countries, namely Australia, United Kingdom, United States, and Canada (4). Existing research indicates that communities, especially priority groups have limited access to evidence-based stigma reduction programs.

## Recommended solutions

Addressing the stigma linked to dementia presents a multifaceted challenge, necessitating a range of interventions across multiple levels, including intrapersonal, interpersonal, community, organizational, and structural dimensions (69) (Table 1). To support these recommendations, our team drew on the social-ecological model (70), ensuring recommendations are culturally sensitive and customized for maximum reach.

**Table 1:** Summary of suggested activities according to target level.

Levels	Activities
Intrapersonal level	Education materials in native languages based on target population literacy levels (workshops, digital material, videos, infographics)
	Working along with their traditional practitioners and bilingual healthcare workers as facilitators for treatment
Interpersonal level	Providing education about dementia to their family carers, community carers
	Community-based habitations
Community level	Educational materials (factsheets, online modules, videos, films, podcasts)
	Working with respective stakeholders to increase community participation in the programs
	Available support groups, helpline, peer support networks
	Community champions: identify and train community leaders, influencers, and volunteers
	Supporting intergenerational family care
Organizational/Institutional level	Curriculum-based training programs for healthcare students that focus on cultural training
	Professional training for healthcare workers about their cultural of these communities
Governmental/Structural level	Legal and policy interventions - anti-discriminatory policies
	Rights-based approaches
	National anti-stigma messaging campaigns
	Funding for CALD- specific dementia services
	Implementing dementia- friendly communities
	Mandatory cultural competency training to healthcare professionals, aged care workers and providers

The social-ecological model, a conceptual framework that emphasizes the interplay of individual, interpersonal, institutional, community, and policy elements in shaping individuals’ experiences (70), has emerged as a pivotal organizational lens in our analysis, specifically focusing on the pervasiveness of dementia stigma within cultural communities. This model offers a structured approach for comprehending how both social and structural determinants contribute to the perpetuation of dementia-related stigma and influence assessment and care strategies in the global context.

Prior research has effectively employed the social-ecological model to explore issues related to cultural communities, their attitudes toward dementia, accessibility to dementia care, and awareness about dementia (71–74). Critically, this model remains dynamic, with constituent elements of this model guiding endeavors aimed at reducing dementia stigma within cultural communities. This guidance can manifest through the development of culturally sensitive interventions or the strengthening of existing practices and approaches, ultimately fostering improved dementia care within diverse cultural settings.

Initially, at the intrapersonal level, strategies should prioritize addressing the unique characteristics of the illness and societal taboos experienced by individuals living with the disease. These programs should prioritize self-help, therapy, and counseling (69). Interventions should be collaboratively developed with target groups to address linguistic and cultural needs. They should be delivered in appropriate and engaging ways, such as utilizing technology to customize messaging and support, such as personalized SMS, or a combination of methods. Tailored initiatives can empower CALD individuals with dementia and their carers by enhancing their understanding of the condition, facilitating symptom management, and promoting timely self-care. Collaboration with traditional practitioners (e.g., Chinese medicine), faith leaders, bilingual healthcare professionals (e.g., General Practitioners) and interpreters trained in dementia-specific conversations within these communities is important to understand their illness through individualized counseling sessions and promote appropriate treatment options (7). This collaboration can foster positive attitudes, thereby reducing the negative effects of stigmatizing attitudes in these communities.

At the *interpersonal level*, it is important for programs to focus on improving care and support for individuals with dementia in accordance with their social and cultural surroundings. In CALD communities, it is important to ensure that care materials are culturally appropriate and acceptable. Additionally, engaging in motivational interviewing and sharing positive stories of individuals with dementia in their native language can be beneficial. However, careful tailoring is required to ensure that messaging, designs and materials resonate with target communities (5). These efforts can result in changes in attitudes, behaviors, and perceptions toward individuals with dementia.

At the *community level*, efforts should focus on reducing stigmatizing attitudes and behaviors among non-stigmatized groups through the implementation of strategies such as education, contact, and advocacy. To achieve this objective, communication strategies that aim to highlight positive narratives and employ motivational interviewing (e.g., through videos and films) customized to suit the literacy levels of specific target groups should be utilized. Providing educational factsheets and challenging negative stereotypes are also potential means of achieving this goal. Additionally, applying a technique that emphasizes favorable instances of resilience, adaptation, and communal assistance can help counteract stigmatization and promote compassion within individuals. The delivery of these measures should be supported by collaboration with relevant stakeholders, including local community leaders, spiritual leaders, community organizations, non-profit organizations, media, networks, community events, social gatherings, and places of worship (such as temples, churches, mosques, etc.). This collaborative approach is likely to facilitate increased participation of these communities in the programs, improve their understanding of dementia, and enhance their access to suitable healthcare services.

At the *institutional/organizational level*, the approaches should focus on mitigating stigma within the structure by employing measures such as cultural training programs (education and contact) to professionals and curriculum-based training for students. These measures should target various groups such as healthcare professionals, community workers, healthcare students, police, counselors, and so on. The implementation of these measures, especially for healthcare professionals, is expected to result in the provision of care that is both clinically safe and culturally sensitive.

Finally, at the *structural/governmental level*, interventions should focus on establishment and implementation of legal, policy, and rights-based structures. In these communities, dementia diagnosis, treatment and care are influenced by cultures, religions, and beliefs. Consequently, it is necessary for health services and health promotion initiatives to consider specific cultural needs to maximize participation and enhance the capabilities of individuals with dementia in these populations. Maintaining cultural appropriateness is an ongoing process that requires consistent engagement with relevant community members and organizations throughout the planning, execution, and assessment phases. This approach is likely to promote cultural values and enhance the effectiveness of interventions.

## Future directions

Future advances are essential in the field of dementia stigma research, particularly within CALD communities. As our understanding of dementia stigma continues to evolve, it becomes increasingly evident that a one-size-fits-all approach is insufficient. To effectively combat stigma within these diverse cultural contexts, a tailored and culturally sensitive approach is essential. The following proposed directions seek to address the limitations in existing tools, evaluation methods, and awareness campaigns, all while acknowledging the role of social determinants of health across the life course. By focusing on these future directions, researchers and practitioners can contribute to reducing stigma and improving the quality of dementia care for CALD communities in a more targeted and impactful manner.

### Development of culturally relevant instrument measuring dementia-related stigma

According to the existing literature, dementia-related stigma evaluation instruments contain inconsistencies, and tools initially designed for HIV/AIDS, cancer, mental health, and chronic diseases were utilized (75). By developing culturally sensitive measurement instruments, future research can yield more accurate and nuanced insights into the prevalence and significance of dementia stigma within specific cultural groups. These instruments should be tailored to account for language, health literacy, and cultural preferences, ensuring that the stigma assessment aligns with the unique characteristics of each community. This approach not only enhances the rigor of the research but also fosters inclusivity and a deeper understanding of the cultural factors influencing stigma.

### Evaluation methods

Effective evaluation techniques are critical in assessing the strengths and weaknesses of community programs aimed at reducing dementia stigma within culturally diverse settings (9). Such evaluations provide valuable insights that can be used to refine and improve programs to better meet the specific needs of target communities. Additionally, collaborative partnerships with the cultural communities are essential to develop interventions grounded in evidence and tailored to their unique requirements (76). This approach fosters the creation of programs that are not only culturally appropriate but also cost-effective, feasible, and scalable, ensuring their long-term impact on reducing dementia-related stigma within diverse communities.

### Life course model of the social determinants of health

The adoption of a life course model to address dementia stigma within culturally diverse communities represents a holistic approach that acknowledges how health and attitudes are shaped throughout a person’s lifetime (77). By targeting various life stages, from early childhood through retirement, future initiatives can focus on changing attitudes, beliefs, perceptions, and behaviors related to dementia (78) as well as explore diverse outcomes [e.g., (79)]. This comprehensive strategy can help reduce stigma-associated issues in culturally and linguistically diverse (CALD) communities while also narrowing health disparities. The potential long-term benefits of this approach make it a promising direction for future efforts to combat dementia stigma among population groups with diverse cultural backgrounds.

## Conclusion

The pervasive stigma of dementia in CALD communities is influenced by cultural beliefs, language barriers, lack of awareness, and the impact of migration. Addressing this stigma is crucial to ensure equitable access to dementia care and support for CALD individuals and their families. Promoting dementia awareness in minority communities through active involvement of targeted groups and emphasizing the retained abilities of individuals with dementia can enhance their quality of life, support caregivers, and improve access to essential services and resources. By implementing culturally sensitive interventions, educational campaigns, and support networks, the stigma can be challenged, and CALD communities can become more inclusive and supportive environments for individuals living with dementia. Finally, collaboration between policymakers, healthcare providers, community organizations, and CALD community members is essential to create a dementia-inclusive society that respects and supports the dignity and well-being of all its members.

## Data availability statement

The original contributions presented in the study are included in the article/supplementary material, further inquiries can be directed to the corresponding author.

## Author contributions

JS: Conceptualization, Supervision, Writing – original draft, Writing – review & editing. AM: Conceptualization, Writing – original draft, Writing – review & editing. JA: Writing – review & editing.
